# Takotsubo Cardiomyopathy and Trauma: The Role of Injuries as Physical Stressors

**DOI:** 10.7759/cureus.27411

**Published:** 2022-07-28

**Authors:** Carlos A Fernandez, Joel R Narveson, Ryan W Walters, Neil D Patel, Jessica M Veatch, Kaily L Ewing, Thomas J Capasso, Viren P Punja, Eirc J Kuncir

**Affiliations:** 1 Trauma Surgery and Critical Care, Creighton University Medical Center, Omaha, USA; 2 Surgery, Creighton University School of Medicine, Omaha, USA; 3 Clinical Research and Evaluative Sciences, Creighton University School of Medicine, Omaha, USA

**Keywords:** physical stressors, national trauma data bank, trauma, stress cardiomyopathy, takotsubo cardiomyopathy

## Abstract

Introduction: Physical stressors are common predisposing factors for takotsubo cardiomyopathy (TTC). However, the role of traumatic injuries in TTC has not been well defined. This study describes the characteristics of TTC in the broad spectrum of traumatic injuries using the information available in the National Trauma Data Bank (NTDB).

Materials and methods: This retrospective study analyzed trauma patients ≥ 18 years old in the NTDB, from 2007 to 2018, with a diagnosis of TTC.

Results: A total of 95 TTC diagnoses were found. The median age was 68 years old (interquartile range: 55-80). Patients were predominantly female (67.4%), white (88.4%), and sustained blunt mechanisms of injury (90.5%). Penetrating trauma was most common in males (16%). Most diagnoses were related to extremity trauma (53.7%), followed by head injury (26.3%). The most common severity scores were Glasgow Coma Scale (GCS) > 13 or < 8, and Injury Severity Score (ISS) < 15 or > 25. Males more commonly presented with GCS < 8 (68%), ISS > 25 (33%), high intensive care unit (ICU) admission rate (77.4%), and mechanical ventilation (51.6%). The median duration of the mechanical ventilation was eight days for both sexes. The ICU length of stay (LOS) was six days with a hospital LOS of nine days and a trend toward a longer LOS in males. The in-hospital mortality rate was 11.7% for both sexes.

Conclusions: TTC in traumatic injuries is common at both ends of the severity spectrum and has different sex distribution. TTC patients are predominantly females and have more commonly extremity trauma than head injury. Males are more severely injured and under mechanical ventilation.

## Introduction

Takotsubo cardiomyopathy (TTC) is a transient ballooning of the left ventricle, predominantly recognized in elderly women with recent significant emotional or physical stress [1-3]. The pathophysiology of the TTC is not fully understood, but evidence suggests that the pathogenesis of the disease is influenced by multiple factors, including the brain-heart axis with over-stimulation of the sympathetic system, microvascular and myocardial tissue metabolism abnormalities, and coronary artery vasospasm [1,3-5]. The diagnosis of TTC is based upon the Mayo Clinic or International Takotsubo Diagnostic Criteria (Appendix) and requires careful differentiation from acute coronary syndrome, given their similar clinical electrocardiogram and enzymatic presentations, yet without obstructive coronary artery disease or plaque rupture [1,2]. Prompt diagnosis of TTC is important to minimize the risk for adverse events, as early diagnosis can inform clinical management and consequently optimize the timing of surgical interventions [6-10]. The spontaneous resolution of left ventricular wall motion abnormalities in TTC usually occurs in hours to weeks, and elective procedures can be delayed until the resolution of the cardiomyopathy [6]. In terms of risk of mortality, TTC has a similar mortality rate compared to patients with acute myocardial infarction but with a more favorable and faster recovery [6].

The diagnostic hallmark of TTC is its association with a stressful event. Although initial triggers were predominately limited to emotional trauma, recent evidence suggests TTC occurs with physical triggers or even in the absence of any recognizable stressors [2,11,12]. Potential physical stressors have been previously described, for example, surgical procedures, severe illnesses, fractures, dobutamine stress tests, electroconvulsive therapy, and cocaine use [1]. Physical triggers may be more common than psychological stressors, and male patients are more often affected by a physical stressor, while women more frequently report an emotional trigger [2,6].

The reported prevalence of TTC varies widely according to the patient population. For example, 1-3% of all patients presenting with clinical manifestation of acute coronary syndrome have TTC, and this increases to 10% if only women are considered [3]. The incidence of TTC has been reported to be between 5.7% and 28.0% in the medical intensive care unit (ICU), whereas in general acute hospitalization, the diagnosis is made in 0.02% of all patients [13,14]. In the trauma literature, TTC has been reported in 10% of patients with traumatic brain injury [15], 18.4% with extreme physical activity or trauma [16], and 15% with perioperative myocardial damage and hip fracture. However, this information on TTC is not related to the trauma population in general and predominantly represents a series of cases involving limited traumas [4,7,10,13,17-19]. This study is the first report assessing the TTC information available from the National Trauma Data Bank (NTDB) to better characterize the disease among the broad spectrum of traumatic injuries.

## Materials and methods

This study was approved a priori by the local institutional review board (#2002373), conforming to the ethical guidelines of the 1975 Declaration of Helsinki. Informed consent was waived due to the retrospective nature of the study and minimal risk as determined by the local institutional review board.

Study population

This study is a retrospective analysis of the NTDB from 2007 to 2018, involving adult trauma patients (≥18 years old) with a diagnosis of TTC. NTDB was selected as it is relevant to the population of interest, offers a large database, and may help limit bias from one hospital or geographic region.

Study variables

Codes described in the Trauma Quality Improvement Program Participant Use File coding system were used to extract our outcome variables. Takotsubo was identified using the International Classification of Diseases, Ninth Revision (ICD-9) and Tenth Revision (ICD-10) Clinical Modification codes 429.83 and I51.81. Patients with acute coronary syndrome or myocardial infarction diagnoses were excluded from our analysis. Unfortunately, given the nature of the NTDB, we could not discern the criteria of the takotsubo ICD-10 diagnostic codes outlined in the manuscript, given the deidentified nature of the data. The NTDB is based upon incidents, for example, a patient could be repeated in the dataset without any way to discern this. Thus, the word "incident" was used rather than "patient." For each trauma incident meeting inclusion criteria, we described the age, sex, race, mechanism of injury, Injury Severity Score (ISS), Glasgow Coma Scale (GCS) score, Abbreviated Injury Scale (AIS) severity per body region, and comorbidity data. We also described the in-hospital mortality rate, ICU length of stay (ICULOS), and hospital length of stay (HLOS).

Statistical analysis

Depending on data distribution, continuous variables are presented as mean ± standard deviation (SD) or median and interquartile range (IQR). Categorical variables are presented as frequency and percentage. Outcomes are presented as estimates with 95% confidence intervals. We stratified baseline characteristics and outcomes by sex with between-sex differences in baseline characteristics compared using the exact Mann-Whitney test for continuous variables and Fisher's exact test for categorical variables. In-hospital death was estimated using a log-binomial regression model. The Kaplan-Meier method was used to evaluate hospital and ICU length of stay as the probability of discharge; between-sex comparisons were evaluated using the log-rank test. SAS version 9.4 (SAS Institute Inc., Cary, NC) was used for all statistical analyses with a two-tailed p < 0.05 used to indicate statistical significance.

## Results

Frequency of reported TTC in the trauma population

**Figure 1:**
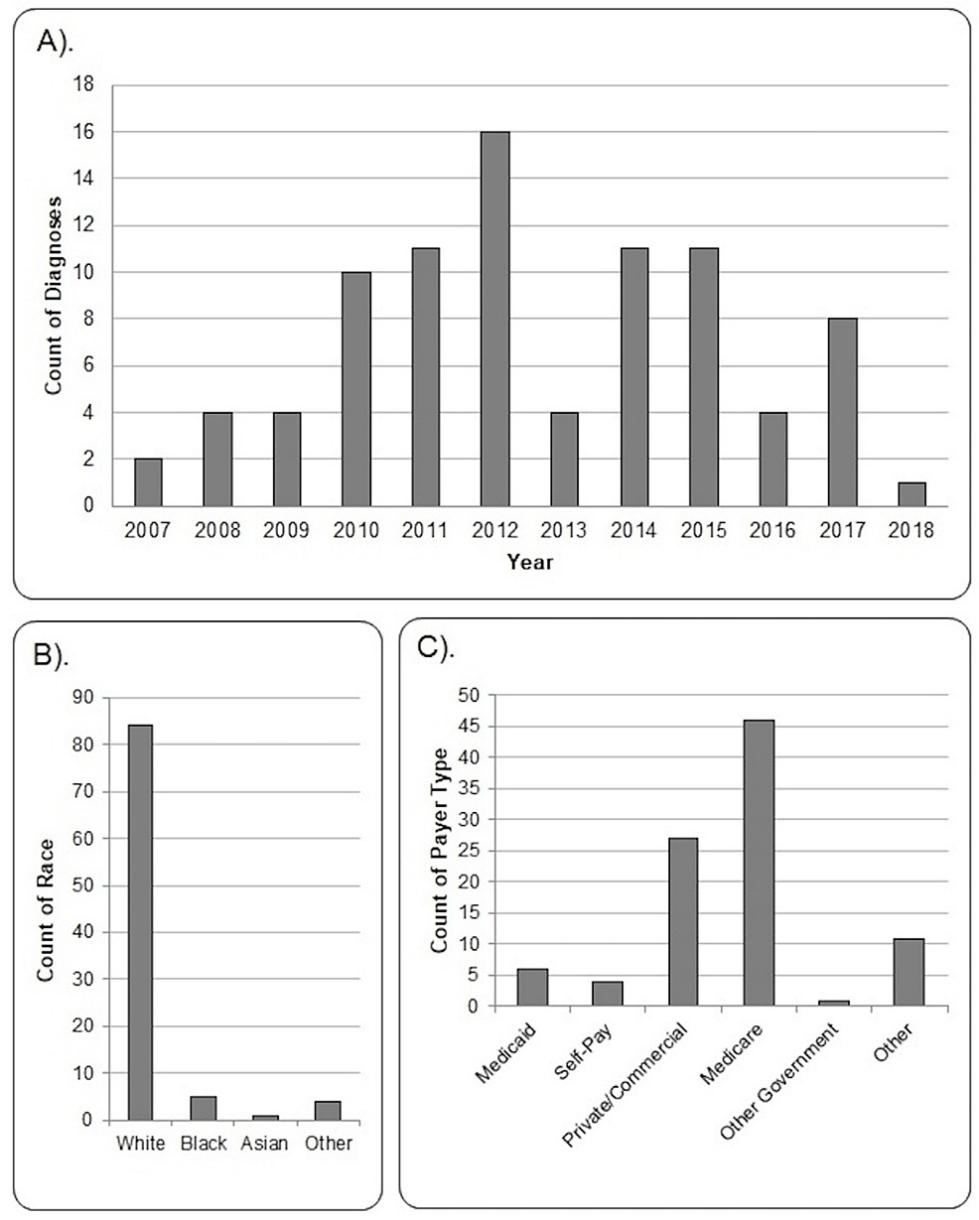
(A) The number of reported TTC diagnoses by year. (B) Count of TTC diagnoses reported by race. One data point was missing from the National Trauma Data Bank. (C) Count of TTC diagnoses reported by primary insurance type. TTC = takotsubo cardiomyopathy.

The NTDB contained 9,782,951 trauma incidents from 2007 to 2018, of which 2,471,525 were admitted to the ICU. There were 95 reported cases with TTC, which represent approximately 0.001% of the NTDB analyzed. The number of TTC diagnoses reported in the NTDB by year is presented in Figure 1.

Basic demographics

The median age was 68 years old (IQR: 55-80) and five incidents in the NTDB were missing ages. Incidents were more likely to be female (n = 64; 67.4%) than male (n = 31; 32.6%). The most common race was white, and most were insured by Medicare or commercial insurance (Figure 1). Hypertension was the most frequent comorbidity, followed by congestive heart failure, chronic obstructive pulmonary disease (COPD), and diabetes (Table 1).

**Table 1:** Reported comorbidities in patients diagnosed with TTC. Data are presented as n (%). Any data that are missing out of the 95 patients are represented in the table. Comorbidity data for substance use, anticoagulant use, myocardial infarction, and mental/personality disorder were collected beginning in 2017. TTC = takotsubo cardiomyopathy; COPD = chronic obstructive pulmonary disease.

	Missing	Statistic
Smoking history	0	10 (10.5)
Substance use disorder	77	1 (5.6)
Steroid use	0	2 (2.1)
Bleeding disorder	0	14 (14.7)
Anticoagulant use	77	5 (27.8)
Alcohol use disorder	0	13 (13.7)
Dependent health status	0	5 (5.3)
Hypertension	0	57 (60.0)
Congestive heart failure	0	24 (25.3)
Myocardial infarction	77	0 (0.0)
Diabetes	0	17 (17.9)
COPD	0	18 (19.0)
Chronic kidney disease	0	3 (3.2)
Mental/personality disorder	77	2 (11.1)
Cerebrovascular accident	0	8 (8.4)

Injury characteristics

The majority of injuries were blunt, with a median ISS of 9 (IQR: 5-16) and GCS of 15 (IQR: 14-15; Figure 2). See Table 2 for details on the mechanism of injury. When injuries were stratified by sex, statistically significant differences were observed for all injury characteristics (Table 3). A blunt mechanism of injury was more common in females compared to males, whereas a penetrating mechanism was more common in males than in females. A GCS > 13 was reported in 80% with 74% being females and a GCS < 8 was reported in 17.4% with 68% being males. ISS was most commonly classified as either <15 (71.1%) or >25 (17.8%). When stratified by sex, ISS was higher in males compared to females globally, with different distributions as females had 26.7% more injuries with <15, while males had 23.3% more with >25. The GCS score distributions were also different by sex, with males having 27.3% more incidents with severe scores, and females with 28.9% more incidents with mild scores.

**Figure 2:**
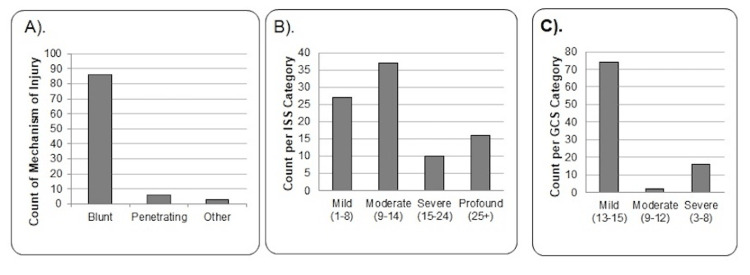
(A) Count of reported mechanisms of injury type for those with TTC. (B) Count of reported ISS by categorical grouping. Six data points were missing from the NTDB in those with TTC. (C) Count of reported GCS scores by categorical grouping. Three data points were missing from the NTDB in those with TTC. TTC = takotsubo cardiomyopathy; NTDB = National Trauma Data Bank; ISS = Injury Severity Score; GCS = Glasgow Coma Scale.

**Table 2:** Mechanism of injury counts.

Mechanism of injury	Count
Fall	59 (62.11%)
Motor vehicle collision	18 (18.95%)
Firearm	3 (3.16%)
Cut/pierce	3 (3.16%)
Other	12 (12.61%)

**Table 3:** Injury characteristics by gender. Data are presented as n (%) or median (interquartile range). ISS = Injury Severity Score; GCS = Glasgow Coma Scale.

	Missing	Overall (N = 95)	Female (n = 64)	Male (n = 31)	P
Mechanism of injury					
Blunt	0	86 (90.5)	62 (96.9)	24 (77.4)	0.005
Penetrating		6 (6.3)	1 (1.6)	5 (16.1)	
Other		3 (3.2)	1 (1.6)	2 (6.5)	
ISS		9 (5-16)	9 (5-14)	14 (9-25)	0.007
Mild (1-8)	5	27 (30.0)	21 (35.0)	6 (20.0)	0.040
Moderate (9-14)		37 (41.1)	27 (45.0)	10 (33.3)	
Severe (15-24)		10 (11.1)	6 (10.0)	4 (13.3)	
Profound (25+)		16 (17.8)	6 (10.0)	10 (33.3)	
GCS		15 (14-15)	15 (14-15)	14 (13-15)	0.002
Mild (13-15)	3	74 (80.4)	55 (90.2)	19 (61.3)	0.002
Moderate (9-12)		2 (2.2)	1 (1.6)	1 (3.2)	
Severe (3-8)		16 (17.4)	5 (8.2)	11 (35.5)	

The majority of incidents had extremity trauma and head injury followed by thorax, spine, face, and abdomen (Table 4). Cumulatively, 53.7% of incidents had extremity injuries compared to 26.3% with head injuries. There were no significant differences between males and females in the distribution of body region injuries. However, the head AIS was significantly higher in males with a trend of higher upper extremity AIS in females. The highest median AIS was 3, predominantly for the lower extremity and head (Table 4).

**Table 4:** Abbreviated Injury Scale body region frequency and severity by sex. Data are presented as n (%) or median (interquartile range). Missing or absent data are presented as "-".

	Count				Severity			
	Overall (N = 95)	Female (n = 64)	Male (n = 31)	P	Overall (N = 95)	Female (n = 64)	Male (n = 31)	P
Other trauma	2 (2.1)	1 (1.6)	1 (3.2)	0.549	-	-	-	-
Head	25 (26.3)	15 (23.4)	10 (32.3)	0.457	3 (1-3)	2 (1-3)	3 (2-5)	0.024
Face	9 (9.5)	4 (6.3)	5 (16.1)	0.146	1 (1-1)	1 (1-2)	1 (1-1)	1.000
Neck	2 (2.1)	0 (0.0)	2 (6.5)	0.104	-	-	-	-
Thorax	11 (11.6)	9 (14.1)	2 (6.5)	0.495	2 (1-4)	2 (2-4)	-	-
Abdomen	5 (5.3)	2 (3.1)	3 (9.7)	0.326	2 (2-4)	-	4 (1-4)	-
Spine	10 (10.5)	6 (9.4)	4 (12.9)	0.724	2 (2-2)	2 (2-2)	2 (2-3)	0.634
Upper extremity	22 (23.2)	13 (20.3)	9 (29.0)	0.437	2 (1-2)	2 (2-2)	1 (1-2)	0.058
Lower extremity	29 (30.5)	23 (35.9)	6 (19.4)	0.153	3 (2-3)	3 (2-3)	2 (1-3)	0.134

In-hospital outcomes

**Figure 3:**
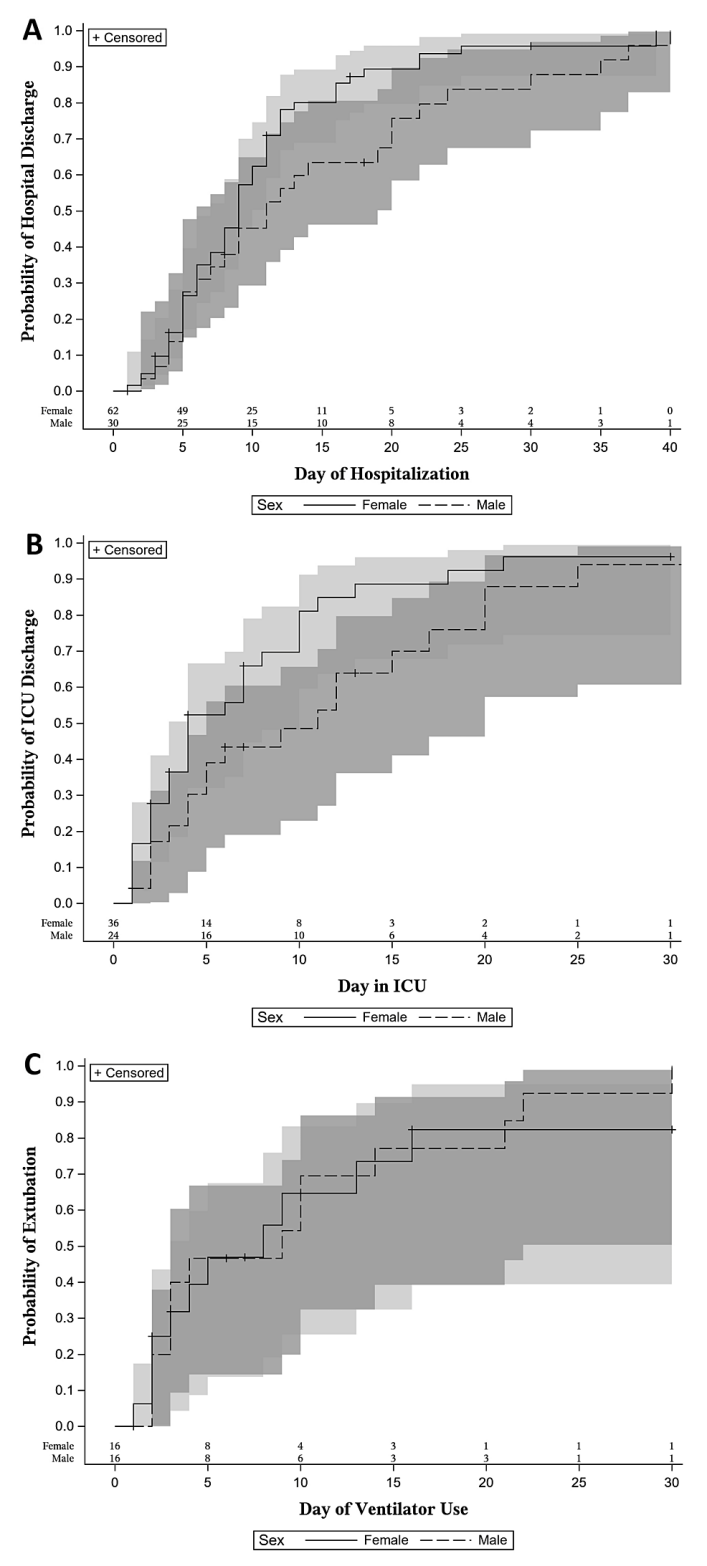
Probability (stratified by sex) of being discharged alive from the hospital (A), being discharged alive from the ICU (B), and being extubated (C). Shaded areas represent 95% confidence intervals.

In-hospital outcomes can be found in Table 5 and were not different between the groups when stratified by sex except for invasive mechanical ventilation status. Males were more commonly admitted to the ICU than females and more likely to be intubated. The HLOS and ICULOS trended longer in males compared to females, although not statistically different. Figure 3 demonstrates the probability of being discharged from the hospital and ICU stratified by sex.

**Table 5:** In-hospital outcomes. Any data that are missing out of the 95 patients are represented in the table. Data are presented as n (%) or estimate (95% CI).

	Missing	Overall (N = 95)	Female (n = 64)	Male (n = 31)	P
All-cause mortality					
In-hospital	1	11.7 (6.7-20.5)	11.1 (5.5-22.6)	12.9 (5.1-32.6)	0.800
ED	4	0	0	0	-
Hospital length of stay	2	9 (8-11)	9 (7-10)	11 (6-19)	0.074
Intensive care unit					
No	0	34 (35.8)	27 (42.2)	7 (22.6)	0.071
Yes		61 (64.2)	37 (57.8)	24 (77.4)	
Length of stay	0	6 (4-10)	4 (3-10)	11 (5-20)	0.076
Invasive mechanical ventilation					
No	0	63 (66.3)	48 (75.0)	15 (48.4)	0.020
Yes		32 (33.7)	16 (25.0)	16 (51.6)	
Duration of mechanical ventilation (days)	0	8 (3-14)	8 (3-30)	9 (3-22)	0.862

Reporting guidelines

We followed the Strengthening the Reporting of Observational Studies in Epidemiology guidelines (Table 6).

**Table 6:** STROBE statement: checklist of items that should be included in reports of cross-sectional studies. STROBE = Strengthening the Reporting of Observational Studies in Epidemiology.

	Item No.	Recommendation	Section/page number
Title and abstract	1	(a) Indicate the study’s design with a commonly used term in the title or the abstract	Title page, Abstract
		(b) Provide in the abstract an informative and balanced summary of what was done and what was found	Abstract
Introduction			
Background/rationale	2	Explain the scientific background and rationale for the investigation being reported	Background
Objectives	3	State specific objectives, including any prespecified hypotheses	Background
Methods			
Study design	4	Present key elements of study design early in the paper	Methods “Study population”
Setting	5	Describe the setting, locations, and relevant dates, including periods of recruitment, exposure, follow-up, and data collection	Methods “Study population” and “Study variables”
Participants	6	(a) Give the eligibility criteria and the sources and methods of selection of participants	Methods “Study population”
Variables	7	Clearly define all outcomes, exposures, predictors, potential confounders, and effect modifiers. Give diagnostic criteria, if applicable	Methods “Study variables”
Data sources/measurement	8	For each variable of interest, give sources of data and details of methods of assessment (measurement). Describe comparability of assessment methods if there is more than one group	Methods “Study population” and “Study variables”
Bias	9	Describe any efforts to address potential sources of bias	Methods “Study population”
Study size	10	Explain how the study size was arrived at	“Statistical analysis”
Quantitative variables	11	Explain how quantitative variables were handled in the analyses. If applicable, describe which groupings were chosen and why	“Statistical analysis”
Statistical methods	12	(a) Describe all statistical methods, including those used to control for confounding	“Statistical analysis”
		(b) Describe any methods used to examine subgroups and interactions	
		(c) Explain how missing data were addressed	
		(d) If applicable, describe analytical methods taking into account of sampling strategy	
		(e) Describe any sensitivity analyses	
Results			
Participants	13	(a) Report numbers of individuals at each stage of the study, e.g. numbers potentially eligible, examined for eligibility, confirmed eligible, included in the study, completing follow-up, and analyzed	Results “Frequency of reported TTC in the trauma population”
		(b) Give reasons for non-participation at each stage	
		(c) Consider the use of a flow diagram	
Descriptive data	14	(a) Give characteristics of study participants (e.g. demographic, clinical, and social) and information on exposures and potential confounders	Results “Basic demographics” and “Injury characteristics”
		(b) Indicate the number of participants with missing data for each variable of interest	
Outcome data	15	Report numbers of outcome events or summary measures	
Main results	16	(a) Give unadjusted estimates and, if applicable, confounder-adjusted estimates and their precision (e.g. 95% confidence interval). Make clear which confounders were adjusted for and why they were included	Results “In-hospital outcomes”
		(b) Report category boundaries when continuous variables were categorized	
		(c) If relevant, consider translating estimates of relative risk into absolute risk for a meaningful time period	
Other analyses	17	Report other analyses done, e.g. analyses of subgroups and interactions and sensitivity analyses	
Discussion			
Key results	18	Summarize key results with reference to study objectives	Discussion and Conclusion
Limitations	19	Discuss limitations of the study, taking into account sources of potential bias or imprecision. Discuss both the direction and magnitude of any potential bias	Discussion “Limitations and strengths”
Interpretation	20	Give a cautious overall interpretation of results considering objectives, limitations, multiplicity of analyses, results from similar studies, and other relevant evidence	Discussion
Generalisability	21	Discuss the generalizability (external validity) of the study results	Discussion “Limitations and strengths”
Other information			
Funding	22	Give the source of funding and the role of the funders for the present study and, if applicable, for the original study on which the present article is based	“Conflicts of interest and source of funding”

## Discussion

In this study, we analyzed the information in the NTDB to expand the association of TTC within the broad spectrum of traumatic injuries. Even though the information available in the NTDB is limited, our analysis is an important first step to identifying epidemiological factors that help better characterize TTC in this particular set of high-risk patients.

Frequency of reported TTC in the trauma population

Trauma patients suffering from physical stressors may be predisposed to a higher incidence of TTC. As we described before, TTC has been reported in 10% of patients with traumatic brain injury [15], 18.4% with extreme physical activity or trauma [16], and 15% with perioperative myocardial damage and hip fracture [17]. However, only 0.001% of trauma incidents in the NTDB reported TTC from 2007 to 2018. This number is very low despite the association between physical triggers and the development of TTC [2,11,12], and it may be related to missed diagnoses, difficulty diagnosing TTC in the trauma population, or underreports of this important comorbidity in the NTDB. The number of diagnoses of TTC displayed over the years might be related to increasing awareness and changes in the diagnostic criteria over time [2] that could explain the up and down trending aspects of the distribution curve. However, a real explanation is unknown to us.

Basic demographics

The median age found for TTC diagnoses in the NTDB was 68 years old, and over two-thirds were female. This finding is consistent with the literature reporting TTC is most common in female patients older than 50 years [1,2,20-22]. The most frequent race reported was white, with insurance coverage by either Medicare or commercial insurance. The literature does report the majority of TTC cases to occur in individuals reporting white race [1,22-25]. Two studies reported generally similar trends for insurance coverage in those diagnosed with TTC [22,25].

The most common comorbidities we found were hypertension, congestive heart failure, COPD, and diabetes, consistent with previous reports [12,21,22,25-27]. However, other studies have reported the most common comorbidities as smoking, alcohol abuse, anxiety states, and hyperlipidemia [24]. The reported prevalence of psychiatric and neurologic disorders in patients with TTC has been around 42.3% and 27%, respectively [6,21]; unfortunately, we could not assess that prevalence due to the high number of missing data on mental/personality disorders in the NTDB before 2017.

Injury characteristics

TTC in trauma patients was more commonly reported with extremity trauma (53.7%) and traumatic head injury (26.3%), followed by thoracic (11.6%), spine (10.5%), face (9.5%), and abdominal injuries (5.3%) with similar sex distribution. This distribution of injuries with TCC closely follows the 2016 NTDB report for injury by AIS body region except for a lower rate of facial injuries [28]. We think this is an interesting finding as the literature previously described TTC in trauma patients predominantly affected by traumatic brain injury [29].

The diagnosis of TTC was almost exclusively made in those with a blunt mechanism of injury for both sexes (90%), although males were more likely to have penetrating trauma than females. The most common mechanisms of injury were falls (62.11%) and motor vehicle collisions (18.95%). Although these mechanisms of injury usually result in head injury, the distribution of TTC incidents follows a different injury pattern, which was one of the most interesting findings of our study.

In terms of severity scores, TTC was reported in all ISS and GCS levels but most commonly in those with GCS > 13 or < 8 and ISS < 15 or > 25, suggesting a higher likelihood of TTC at both ends of the severity scores. Males seemed to be more severely injured as they had lower GCS and higher ISS scores than females (GCS < 8; male: 68%; ISS > 25; male: 62%).

In contrast to a recent systematic review of case reports and cohort studies showing TTC predominantly in head injury incidents with an average GCS of 5 [29], the analysis of the NTDB data shows TTC in trauma patients predominantly with GGS > 13, more often with extremity trauma and with differences in sex distribution on ISS, GCS levels, and rate of mechanical ventilation. This discrepancy in frequency suggests that TTC in the trauma population may be related to high levels of activation of the brain-heart axis at both ends of the severity spectrum where emotional and physical stressors play different roles in the pathogenesis of the disorder.

In-hospital outcomes

The in-hospital mortality rate reported in the NTDB was 11.7%. The median HLOS was nine days (IQR: 5-12), and the median ICULOS was four days (IQR: 2-10), with a trend toward longer hospital and ICU length of stay in males. The majority of the patients were admitted to the ICU, and 33.7% were mechanically ventilated. Among ventilated patients, TTC was significantly more common in males with a median duration of mechanical ventilation of four days (IQR: 2-10) without sex difference.

Our findings for in-hospital outcomes in the trauma population differ compared to the existing literature. The in-hospital mortality rate in the NTDB associated with TCC was higher compared to reported rates of 1.0-6% across various populations [6,14,22,23,25,27], which includes the 4.39% overall mortality rate of trauma patients, according to the NTDB report in 2016 [28]. The 11.7% mortality rate in trauma patients with TTC may be related to the higher number of patients with ISS > 25 (17.8%), as the mortality rate in trauma increases with higher ISS levels. However, if we compare the 22.6% mortality rate of trauma patients with myocardial infarction [30], the overall mortality rate of trauma patients with TCC is lower.

The rate of mechanical ventilation was higher than previously reported rates of 3.6-15% [22,24,25]. The HLOS exceeded the median HLOS of three days (IQR: 2-5) reported in patients > 65 years of age from another national database [23]. However, Syed et al. (2020) reported similar HLOS if the cardiogenic shock was present, as patients had a higher rate of mechanical ventilation of 65% and an in-hospital mortality rate of 23% [22].

Limitations and strengths

First, this is a retrospective study and thus is limited in the ability to make inferences about the data presented. Second, as the data are from the NTDB, and these data are abstracted from the medical records by a variety of trauma registrars, some data may be inaccurate due to varying interpretations of coding. Third, the diagnosis of TTC goes by many names, although every attempt was made to be thorough in extracting relevant diagnoses from the NTDB, diagnoses could have been missed. Fourth, given the nature of the NTDB, data points were missing. This is either due to the NTDB required variables changing from year to year or the data not being submitted to the NTDB from a trauma center. Fifth, head injuries and traumatic brain injury were not specifically isolated from the AIS coding; however, the number of incidents with traumatic brain injury is equal to or less than the number with head injuries. Sixth, relapses in TTC are indistinguishable in the NTDB and could be represented in our dataset, yet, this is unlikely to affect the main conclusions. Finally, a stronger analysis could be done by comparing TTC versus non-TTC trauma patients in future studies. The most notable strength of this study is the utilization of a national database, which expands its external validity.

## Conclusions

TTC in patients with traumatic injuries is common at both ends of the severity spectrum and affects predominantly females with blunt extremity trauma more than with head injury. TTC in trauma seems to have a higher mortality rate than TTC in non-trauma patients and trauma patients without TTC. Differences in sex distribution show males as more severely injured than females and with a higher rate of ICU admission and mechanical ventilation, and a trend toward longer ICULOS and HLOS.

Although it is not surprising to see more TTC diagnoses in females and males having more severe trauma injuries than females, as is the trend in trauma, it is novel to find a pattern of severity distribution of TTC affecting both genders with a higher than usual mortality rate and extremity body region as the most common physical stressors rather than the brain. This study is the first systematic description of TTC in relation to traumatic injuries in the whole trauma population. Given the nature of the NDTB as a national database, we think this study provides the strength of generalizability.

## Appendices

Takotsubo diagnostic criteria

**Table 7:** Takotsubo diagnostic criteria: International and Mayo Clinic Criteria

International Takotsubo Diagnostic Criteria		Mayo Clinic Criteria
"1. Patients show transient left ventricular dysfunction (hypokinesia, akinesia, or dyskinesia) presenting as apical ballooning or midventricular, basal, or focal wall motion abnormalities. Right ventricular involvement can be present. Besides these regional wall motion patterns, transitions between all types can exist. The regional wall motion abnormality usually extends beyond a single epicardial vascular distribution; however, rare cases can exist where the regional wall motion abnormality is present in the subtended myocardial territory of a single coronary artery.		“1. Transient hypokinesis, akinesis, or dyskinesis of the left ventricular mid segments with or without apical involvement; the regional wall motion abnormalities extend beyond a single epicardial vascular distribution; a stressful trigger is often, but not always present.
2. An emotional, physical, or combined trigger can precede the takotsubo syndrome event, but this is not obligatory.		2. Absence of obstructive coronary disease or angiographic evidence of acute plaque rupture.
3. Neurologic disorders (e.g. subarachnoid hemorrhage, stroke/transient ischaemic attack, or seizures), as well as pheochromocytoma, may serve as triggers for takotsubo syndrome.		3. New electrocardiographic abnormalities (either ST-segment elevation and/or T-wave inversion) or modest elevation in cardiac troponin.
4. New ECG abnormalities are present (ST-segment elevation, ST-segment depression, T-wave inversion, and QTc prolongation); however, rare cases exist without any ECG changes.		4. Absence of pheochromocytoma myocarditis”
5. Levels of cardiac biomarkers (troponin and creatine kinase) are moderately elevated in most cases; significant elevation of brain natriuretic peptide is common.		
6. Significant coronary artery disease is not a contradiction in takotsubo syndrome.		
7. Patients have no evidence of infectious myocarditis.		
8. Postmenopausal women are predominantly affected."		
The above text is quoted from the International Takotsubo Diagnostic Criteria from Ghadri JR, Wittstein IS, Prasad A, et al. International Expert Consensus Document on Takotsubo Syndrome (Part I): Clinical Characteristics, Diagnostic Criteria, and Pathophysiology. Eur Heart J. 2018;39(22):2032-46. 10.1093/eurheartj/ehy076		The above text is quoted from Prasad A, Lerman A, and Rihal CS. Apical ballooning syndrome (Tako-Tsubo or stress cardiomyopathy): a mimic of acute myocardial infarction. American Heart Journal. 2008;155(3):408-417. 10.1016/j.ahj.2007.11.008
